# Designing a Broadband Pump for High-Quality Micro-Lasers via Modified Net Radiation Method

**DOI:** 10.1038/srep38576

**Published:** 2016-12-07

**Authors:** Sergey Nechayev, Philip D. Reusswig, Marc A. Baldo, Carmel Rotschild

**Affiliations:** 1 Department of Mechanical Engineering and Russell Berrie Nanotechnology Institute, Technion-Israel Institute of Technology, Haifa 32000, Israel; 2 Department of Electrical Engineering and Computer Science, Massachusetts Institute of Technology, 77 Massachusetts Avenue, Cambridge, MA 02139, USA

## Abstract

High-quality micro-lasers are key ingredients in non-linear optics, communication, sensing and low-threshold solar-pumped lasers. However, such micro-lasers exhibit negligible absorption of free-space broadband pump light. Recently, this limitation was lifted by cascade energy transfer, in which the absorption and quality factor are modulated with wavelength, enabling non-resonant pumping of high-quality micro-lasers and solar-pumped laser to operate at record low solar concentration. Here, we present a generic theoretical framework for modeling the absorption, emission and energy transfer of incoherent radiation between cascade sensitizer and laser gain media. Our model is based on linear equations of the modified net radiation method and is therefore robust, fast converging and has low complexity. We apply this formalism to compute the optimal parameters of low-threshold solar-pumped lasers. It is revealed that the interplay between the absorption and self-absorption of such lasers defines the optimal pump absorption below the maximal value, which is in contrast to conventional lasers for which full pump absorption is desired. Numerical results are compared to experimental data on a sensitized Nd^3+^:YAG cavity, and quantitative agreement with theoretical models is found. Our work modularizes the gain and sensitizing components and paves the way for the optimal design of broadband-pumped high-quality micro-lasers and efficient solar-pumped lasers.

On-chip applications for sensing^1^,^2^, non-linear optics^3^,^4^ and optical communication^5^,^6^ require high-quality factor (high-Q), micro-lasers. Also solar-pumped lasers^7^ (SPLs) have similar demands due to the low solar flux density. Owing to the ultra-high transparency of a gain media and short cavity length of such lasers, the pump must propagate for many cavity cycles before being absorbed. Therefore, the coupling of non-resonant light to high-Q micro-lasers is inefficient. For SPLs^8^,^9^,^10^, in addition to the mode coupling losses, the poor spectral overlap between the sun and the laser gain medium leads to high solar concentration at threshold, low slope efficiency and the need for solar tracking and active cooling^8^,^9^,^10^,^11^,^12^,^13^,^14^. Cascade energy transfer (CET) is a concept in which the absorption and emission spectra of materials form an energetic cascade^15^,^16^,^17^,^18^,^19^,^20^,^21^. CET pump schema enables broadband pumping of high-Q cavities^22^ and SPLs that operate at record low solar concentrations^23^. Optimization of the radiative transfer in CET pump schema is rather challenging endeavor, requiring complicated numerical methods. Coherent methods are inapplicable due to the incoherent nature of excitation. Alternatively, Monte Carlo stochastic approach^24^ is utilized to analyze an incoherent photon transport. However, if the optical path is large in the considered configuration, a calculation for even a single point of a parameter space is time-consuming. Moreover, such methods don’t provide a physical intuition on the involved parameters. In this paper, we develop a theoretical model based on modified net radiation method^25^ that includes a pump and CET sensitizer for planar waveguide^26^,^27^ micro-lasers. This simplistic approach may also encompass CET sensitized micro-lasers in different geometries. Our model is implemented for solar-pumped lasers and for the specific configuration of Nd^3+^:YAG cavity under ideal sensitization. Finally, we present our experimental observations of energy transfer from an organic sensitizer AlQ_3_:DCJTB(2%):Pt(TPBP)(4%)^28^ to an Nd^3+^:YAG cavity, which shows excellent conformity with the net radiation model’s prediction. It is revealed that such a sensitizer enables SPL to operate under non-concentrated sunlight, but the slope efficiency is limited to 0.53% due to optical losses. We discuss the advantages of such a generic and modular method for developing broadband-pumped high-quality micro-lasers.

## Results

**Figure 1:**
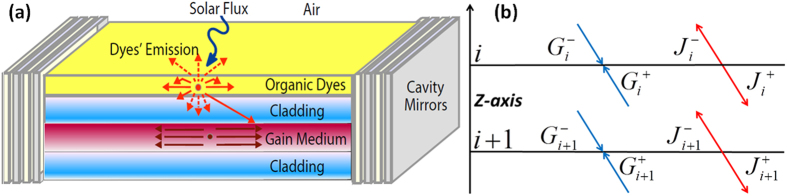
(**a**) A concept device: The pump light is absorbed by a layer of luminescent dyes and is re-emitted into the waveguide, and a fraction of this luminescence is captured in the structure. The captured photons are absorbed by the gain media or reabsorbed by the sensitizer. (**b**) A schematic of radiation net transfer at interfaces *i, i* + *1*. Radiation at each interface on either side is modeled as a sum of incoming and outgoing intensities that impinge at a specific angle, polarization and frequency.

A schematic of the CET sensitized micro-laser in the slab configuration is shown in Fig. 1a. Incident light is absorbed by a layer of sensitizer and is then re-emitted as luminescence. As in the operation of a luminescent solar concentrator (LSC)^28^,^29^,^30^,^31^, a fraction of the emitted photoluminescence is trapped within the waveguide formed by the optical gain media and its sensitizer coating^30^. Light propagating in the waveguide structure is subject to two competitive processes: the absorption in the gain media and the self-absorption in the sensitizer layer itself. Low power threshold *P*_*th*_ micro-lasers must have a small mode volume *V* because *P*_*th*_ is proportional to the mode volume *V* divided by the *Q*-factor: 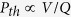, the *Q*-factor being the ratio between the stored energy in the cavity to the energy dissipated per oscillation cycle. Lowering the mode volume means reducing the cavity thickness for the planar waveguide, but this change lowers the cavity absorption at the sensitizer emission wavelength, which must overcome the sensitizer self-absorption for adequate pumping.

Consider a sensitizing material with absorption constant 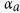 at pump wavelength. Efficient pump absorption requires that the sensitizer layer thickness must be 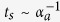, which defines the self-absorption at the sensitizer emission wavelength to be 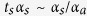, where *α*_*s*_ is the sensitizer self-absorption constant at its emission wavelength. For effective pumping of the gain medium the sensitizer emission wavelength must match the gain media peak absorption coefficient *α*_*g*_. Moreover, cavity absorption must overcome the sensitizer self-absorption, i.e.,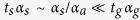, where *t*_*g*_ is the thicknesses of the gain medium. Consequently, while low power threshold *P*_*th*_ demands small *t*_*g*_, effective CET requires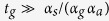. Optimization of this tradeoff between *P*_*th*_ and the CET pumping efficiency is the key to designing an effective CET sensitizer for broadband-pumped high-quality micro-lasers.

We utilize the modified net radiation method^25^, which is a convenient tool for addressing incoherent light absorption (see the Supplementary section I for details on absorption) in planar stratified media, to calculate the profile of the pump light absorption in the sensitizer and the sensitizer’s luminescence absorption in the gain media. In our model, the solar-pumped micro-laser consists of *N* parallel layers (Fig. 1b), i.e., *N* + *2* geometric regions with semi-infinite free-space above the upper interface and below the lower interface. The indices *i* = *0* and *i* = *N* correspond to the upper and lower interfaces, respectively. Without loss of generality we consider a stratified structure with only two absorbing and emitting layers – a sensitizer and a gain medium. In this structure the sensitizer is assumed to be the 1^st^ layer of the micro-laser, and the gain medium is the 3^rd^. Nevertheless, the presented formalism is capable of processing an arbitrary amount of active and passive layers, such as multiple sensitizers with different optical properties on both sides of the structure. Additionally, one may introduce a high-reflectivity mirror on the distal bottom surface of the structure to double pump propagation length within the sensitized micro-laser, and hence pump absorption. A combination of these methods may be utilized for optimal performance. Consider the media enclosed between planes *i* and *i* + *1,* as depicted in Fig. 1b. At the *i*^*th*^ plane, the outgoing and incoming intensities are designated as 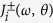 and 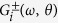, respectively, where *ω, θ* stand for the angular frequency and angle of incidence, respectively. Planar systems have axial symmetry, and therefore, *θ* measured in any layer defines the angle in all of the other layers by Snell’s law. The sign “+” defines intensity components that are situated in the medium above the interface and the sign “−” defines them when situated in the medium below the interface. In addition, in each medium, the incoming and outgoing intensity components are connected by the equations 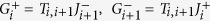 via the transmittance 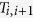 of the layer between planes *i* and *i* + *1*. The transmittance is given by the Beer-Lambert Law 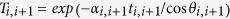, where 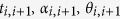 are the thickness, absorption constant and propagation angle in the medium between the planes *i* and *i* + *1*, respectively. The intensity at each interface satisfies 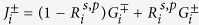, where 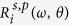, is the Fresnel reflectance for *s, p* waves. The boundary conditions for pump absorption are the incident sunlight from only the upper side, i.e.,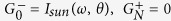, where 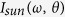 is the solar flux. These linear sets of equations are solved for 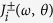 to calculate the absorption in each layer,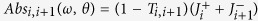. Normal incidence is assumed for a micro-laser that is pumped with a low solar concentration, i.e., 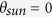. The frequency-dependent absorption is integrated over the solar spectrum to give the total absorbed photon flux in the sensitizer.

After the excitation profile within the luminescent **sensitizing layer** is known, the modified boundary conditions for the luminescence light absorption in the **gain media** can be defined. Assuming that there is no direct pumping of the gain media, then no light is impinging on the micro-laser from either side, i.e.,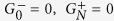; instead, the light is generated within the sensitizer layer. The worst case approximation would be to assume that the luminescent light is uniformly emitted (see Supplementary section II for details on absorption of luminescence and section III for details on non-uniform emission) in the immediate vicinity of the top surface, which enhances the calculated self-absorption. Therefore, the equations for 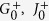 are modified to account for the excitation: 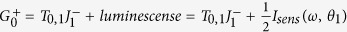, 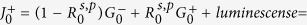 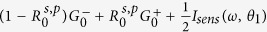, where 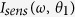 stands for frequency weighted emission of the sensitizer with a uniform distribution inside the sensitizer layer; and 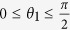. Integration on the emission of the sensitizer, 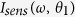, over the frequency and angles 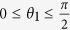 in the sensitizer (see Supplementary section IV for modeling the complex angle of incidence) corresponds to the absorbed solar photon flux multiplied by the sensitizer quantum efficiency 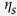. To obtain the photon flux absorbed in the gain media, 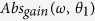 (per angle, per frequency, per polarization), the modified equations are solved for 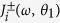. Next, we integrate 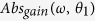 over the sensitizer emission frequencies and for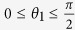. The contributions of the *s* and *p* polarizations are added. The second-order effects of self-absorption and re-emission are also included in the calculation and are modeled as a sum of infinite series of absorption-emission events in the sensitizer, the same as for the LSCs^30^ (see the Supplementary section V).

**Figure 2:**
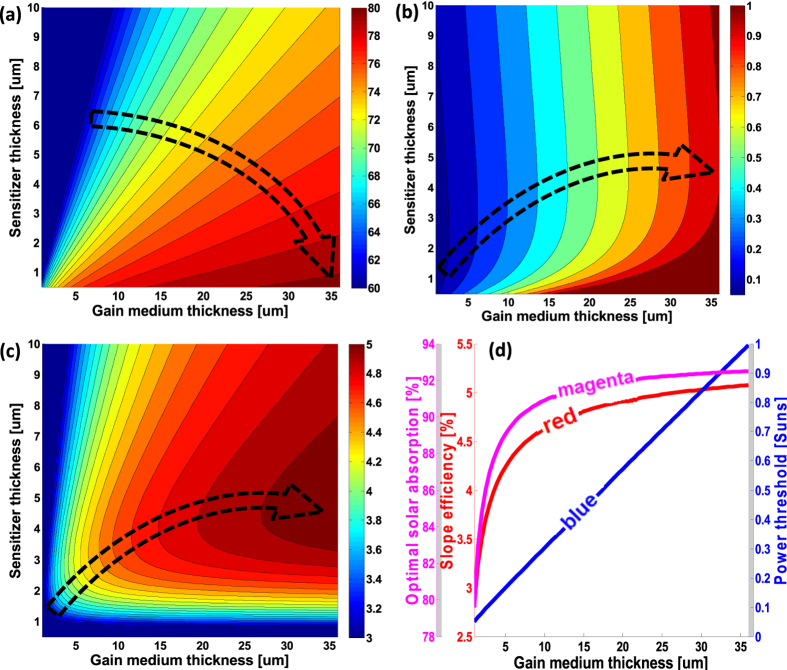
(**a**) The energy transfer between the sensitizer and the gain media is depicted as a fraction of the emitted photoluminescent photons that are absorbed by the gain medium. (**b**) Required solar concentration at the lasing threshold. (**c**) Slope efficiency when the output coupler loss is matched with material losses. Black dotted arrows point to the direction of growing values. (**d**) Slope efficiency (red line, left red axis), solar concentration at threshold (blue line, right blue axis) and optimal absorption (magenta line, left magenta axis) per cavity thickness with the optimal sensitizer layer.

As an example for the above analysis, we examine an ideally sensitized 1% at. Nd^3+^:YAG SPLs. Nd^3+^:YAG has main absorption line at 808 nm^32^ and lasing wavelength *λ*_*L*_ = 1064^32^,^33^,^34^,^35^. Therefore an ideal sensitizer for Nd^3+^:YAG has its emission centered at 808 nm, unity quantum efficiency 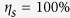 and absorption cutoff at around *λ*_*a*_ = 710 nm, taking into account typical sensitizer Stokes’ shift. The ratio of the sensitizer absorption constants at the absorption band, *α*_*a*_, to its self-absorption constant, *α*_*s*_, assumed to be on the same scale as 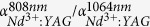, where 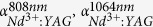 is the absorption constant of Nd^3+^:YAG at 808 nm and its distributed loss constant at 1064 nm, respectively. Hence, we define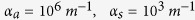, where the last value is approximately the same as the Nd^3+^:YAG absorption coefficient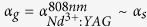. Based on the above estimation the effective pumping regime is reached for cavities much thicker than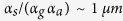. This is in contrast for the required thin cavity supporting low *P*_*th*_. The lasing cavity is assumed to be *l* = 1 cm in length with an output coupler mirror reflectivity that matches the roundtrip cavity material loss of 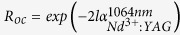. In this case, half of the total power given off by the Nd^3+^ atoms due to the stimulated emission is coupled out of the laser^36^, and the other half is lost owing to material losses. Figure 2a depicts the energy transfer efficiency between the sensitizer and the gain media. This depiction is accomplished by calculating the fraction of photons absorbed in the gain media normalized to the total emitted photons by the sensitizer, which is calculated for various sensitizer and gain media thicknesses. A thicker sensitizer layer means that a larger fraction of the pump is absorbed, but a smaller fraction of photons reaches the gain media owing to the rise in the self-absorption at the sensitizer. For a sufficiently thick cavity and a sufficiently thin sensitizer layer, i.e., 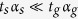, the ratio of absorbed photons in the gain media reaches the value of the captured photons in the waveguiding structure of 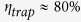
30, which is limited only by Snell’s law, but such a regime in not optimal owing to poor pump absorption. The tradeoff between the absorption and self-absorption leads to a distinct optimum sensitizer thickness when calculating the solar concentration at the lasing threshold (Fig. 2b), which is defined as 
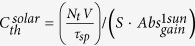
. Here, 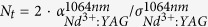 is the population inversion of the cavity at threshold, 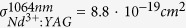 is the emission cross-section at the lasing wavelength, and factor two arises from equating the output coupler and cavity material losses. *V* is the volume of the gain media, *S* is the surface area of the device, *τ*_*sp*_ = 230 *μsec* is the Nd^3+^:YAG fluorescence lifetime, and 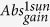 is the photon flux absorbed in the gain media, when the sensitizer is pumped by non-concentrated solar radiation. As seen, the optimal sensitizer thickness is defined by the gain media thickness via the absorption tradeoff. Figure 2c depicts the laser slope efficiency for various thicknesses, and it also shows the optimum value due to the competition in absorption. The slope efficiency is defined as 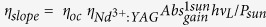, where 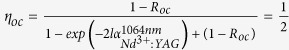 is the useful output, i.e., the fraction of the total power that is coupled out of the laser and is adjusted via the output coupler reflectivity^36^. Here, 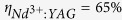 is the Nd^3+^:YAG quantum efficiency, 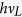 is the energy of the photon at the lasing frequency 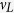, and 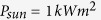 is the solar power per unit area. In contrast to conventional lasers, in which full absorption is desired, here the optimum sensitizer thickness does not correspond to full solar absorption and scales as 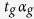. The ratios 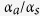 and 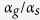 define the sensitivity of the optima and strongly influence the fraction of the absorbed photons in the gain medium. Figure 2d analyzes the optimal conditions for the SPLs, i.e., the sensitizer layer is set at the optimal value for each gain medium thickness. The optimal pump absorption is defined by the optimal sensitizer layer thickness and is shown in magenta (magenta solid line, left magenta axis). The minimal solar concentration at threshold is shown to be less than one sun for a gain media that is thinner than 35 *μm* (blue line, right blue axis). The maximal slope efficiency of SPL (red line, left red axis) is at the optimal absorption value (optimal sensitizer thickness); it increases with the gain media thickness because the thicker gain media allows a thicker sensitizer and consequently a higher pump absorption. Thus, ideally sensitized Nd^3+^:YAG-based SPLs could operate at a non-concentrated solar pump with a 5% slope efficiency. The maximal slope efficiency saturates as the optimal sensitizer thickness reaches full pump absorption.

Notably, in the thin planar waveguide, the lasing mode tail overlaps with the high absorption region of the sensitizer, which significantly affects the resonator Q-factor. Therefore, it is constructive to induce a spatial separation between the sensitizer and the gain medium layers. In contrast to near-field sensitization, radiative energy transfer allows avoiding this negative effect by introducing lossless cladding with an intermediate refractive index between the sensitizer and gain medium (as shown in Fig. 1a), which effectively confines the lasing mode in the low loss region and increases the Q-factor to the value of the unperturbed cavity. The results presented in Fig. 2 are relevant for this case as they assume lossless cladding (see Supplementary section VI for the discussion).

**Figure 3:**
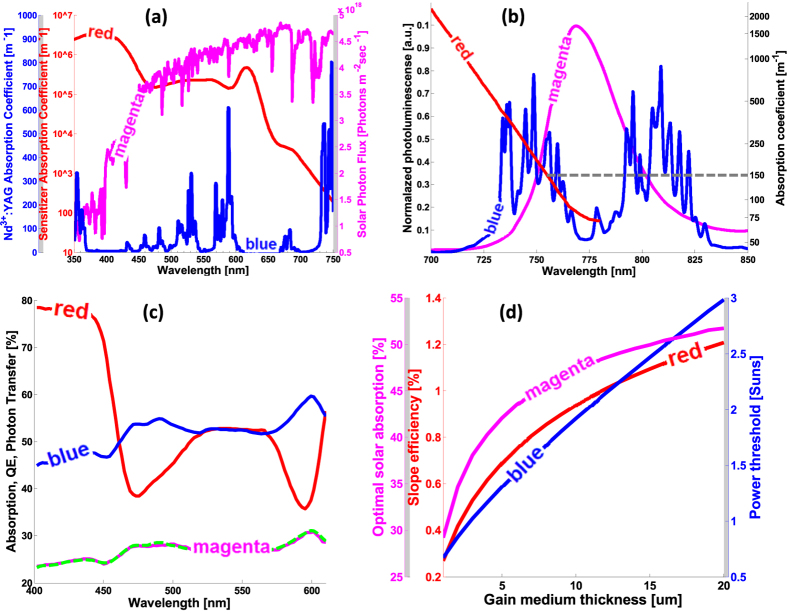
(**a**) Nd^3+^:YAG (blue line, left blue axis) and organic sensitizer (red line, left red logarithmic axis) absorption constant in the visible spectral range overlaid with the solar flux (magenta line, right magenta axis). (**b**) Pt(TPBP) luminescence spectrum (magenta line, left axis) overlaps the Nd^3+^:YAG absorption coefficient (blue line, right logarithmic axis). Red solid line and gray dashed line depict the sensitizer measured and the approximated self-absorption constants, respectively (right logarithmic axis). (**c**) Experimentally measured absorption of the 3.2-μm-thick AlQ_3_:DCJTB(2%):Pt(TPBP)(4%) layer (red solid line) and its external quantum efficiency (blue solid line). The magenta solid and green dashed lines present the measured and predicted ratios between the Nd^3+^ emission rate and the initially absorbed photon rate for the 3.2-μm-thick sensitizer deposited on the 750-μm-thick Nd^3+^:YAG slab cavity. (**d**) Predicted slope efficiency (red line, left red axis), solar concentration at threshold (blue line, blue right axis) and optimal absorption (magenta line, left magenta axis) per cavity thickness with optimal sensitizer thickness.

As experimental validation, we apply our theory to an organic sensitizer and Nd^3+^:YAG planar cavity. The sensitizer is composed of a combination of dyes AlQ_3_:DCJTB(2%):Pt(TPBP)(4%)^28^ that harvest solar radiation between 350 nm and 650 nm. Figure 3a shows the absorption coefficient of this sensitizer (red line, left red logarithmic axis), overlaid with solar photon flux (magenta line, right magenta axis). Nonradiative (near field) energy transfer^15^,^16^,^17^,^18^ from the AlQ_3_ to the DCJTB continuing to the Pt(TPBP) with close to unity efficiency allows to reduce the concentration of the emitting dye - Pt(TPBP), which results in its absorption constant being orders of magnitude higher than for Nd^3+^:YAG in the visible spectrum (Fig. 3a, blue line, left blue axis) while maintaining low self-absorption, below the Nd^3+^:YAG absorption (Fig. 3b, red solid line, right logarithmic axis). The Pt(TPBP) has an emission peak at 780 nm with a full-width half-maximum of 50 nm (Fig. 3b, magenta line, left axis). This emission overlaps with the Nd^3+^:YAG absorption lines, as shown in the blue solid line in Fig. 3b (right logarithmic axis). The value of the Pt(TPBP) self-absorption coefficient that is assumed in our simulations is the maximal value of the experimental data and approximated self-absorption (Fig. 3b, red solid and gray dashed lines, respectively, right logarithmic axis). We have grown a 3.2-*μm*-thick sensitizer layer via thermal deposition on a glass slide to characterize the absorption and external quantum efficiency (Fig. 3c, red and blue lines, respectively), under incoherent continuous-wave excitation at various wavelengths. To measure the pumping efficiency, which is the ratio of the absorbed pump photons to the photons emitted by the Nd^3+^, the same sensitizing layer was grown on a 750-*μm*-thick Nd^3+^:YAG slab. As shown in Fig. 3c, magenta solid line, approximately 27% of the absorbed photons are transferred to Nd^3+^ emission. This value is due to the quantum efficiency of Nd^3+^:YAG, 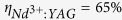
34, the sensitizer quantum efficiency 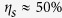 and the trapping efficiency 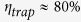
30. Comparing these results to the theoretical model, we note that for the 750-*μm*-thick waveguide and 3.2-*μm*-thick sensitizer, the condition 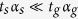 is satisfied since 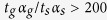. For such a case, the energy transfer efficiency between the sensitizer and the gain media is at the maximal theoretical value of 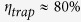, and therefore, the overall photon transfer efficiency is 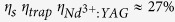. Using the theoretical model with the experimental data on the quantum efficiency (Fig. 3c, blue line) results in the predicted energy transfer per wavelength to the Nd^3+^ emission. This prediction is depicted by the green dashed line in Fig. 3c and shows conformity with the measured values (Fig. 3c, magenta solid line).

Based on our simulations and experimental data, such a sensitizer makes it possible to construct SPLs that operate under non-concentrated solar illumination. As shown in Fig. 3d, the solar threshold (blue line, right blue axis) reaches a non-concentrated condition at a cavity thickness of 3 *μm* (blue line below 1 sun). Unfortunately such cavities are not readily available and the available sensitizers saturate at intensities of *P*_*sun*_ and above (see Supplementary section VII) and an actual device yet to be demonstrated. Moreover, in this case, the slope efficiency is only 0.53% (Fig. 3d, red line, left red axis). Such a low slope efficiency is a result of few factors: **i.** the self-absorption assumed in our simulations (gray dashed line in Fig. 3b), **ii.** non-unity quantum efficiency of the gain media and sensitizer (blue solid line in Fig. 3c), and **iii.** a mismatch between the sensitizer emission and peak Nd^3+^ absorption line (magenta and blue solid lines in Fig. 3b), which results in a disadvantageous ratio of sensitizer self-absorption and Nd^3+^:YAG absorption at the sensitizer emission wavelength. These losses affect the absorption tradeoff and set the optimal sensitizer absorption at the very low value of only 38% (Fig. 3d, magenta line, left magenta axis), which becomes the main limitation of the overall efficiency.

Apart from necessary improvements in all the aforementioned parameters of the sensitizer, another root may be taken to optimize SPLs performance. Namely, a rise in efficiency is expected with a development of a CET sensitizer with emission wavelength in the 940 nm region. Such sensitizer would allow harvesting solar photons in broader spectral range and would make feasible efficient pumping of a variety of Yb-doped gain media.

## In Conclusion

we present a generic theoretical framework for designing a broadband pump schema for micro-lasers and SPLs based on the net radiation method. The formalism reveals that the interplay between the sensitizer self-absorption and cavity absorption defines the optimal value for the pump absorption. This finding is in sharp contrast to conventional lasers in which full absorption is desired. The presented theoretical approach is generic and modular, which allows individual explicit optimization of each component of the broadband-pumped high-Q lasers, providing the guidelines for optimal configuration. Beyond on-chip micro-lasers and SPLs, this method can be useful for other illuminating devices such as LSCs.

## Methods

The 750-μm-thick Nd^3+^:YAG slab waveguide was formed through conventional polishing techniques from commercial laser rods with a diameter of 3 mm and length of 43 mm, with λ = 1064 nm antireflection coatings on both faces. A 3.2-μm-thick film of AlQ_3_:DCJTB(2%):Pt(TPBP)(4%) was thermally evaporated on the Nd^3+^:YAG slab, and layers of various thicknesses were thermally deposited on microscopic glass slides for preliminary experiments. The AlQ_3_:DCJTB(2%):Pt(TPBP)(4%), Nd^3+^:YAG and Nd^3+^:YAG with AlQ_3_:DCJTB(2%):Pt(TPBP)(4%) films were excited with monochromatic light. The monochromatic light was generated using a tungsten-bulb monochromator with a spectral resolution of 3.5 nm full-width at half maximum and a mechanical chopper. The sample photoluminescence was measured with a lock-in amplifier and a spectrally calibrated photodetector, and long wavelength filters with a cutoff at λ = 850 nm were used to discriminate between Nd^3+^:YAG and AlQ_3_:DCJTB(2%):Pt(TPBP)(4%) emission in the integrating sphere. An Acton spectrometer with a grating of 1000 grooves per mm and a calibrated Newport photodetector and neutral density filter were used in all of the spectral measurements. All of the simulations for solving the net radiation method equations were performed on a desktop computer with 32 GB RAM and are detailed in the supplementary materials.

## Additional Information

**How to cite this article**: Nechayev, S. *et al*. Designing a Broadband Pump for High-Quality Micro-Lasers via Modified Net Radiation Method. *Sci. Rep.* **6**, 38576; doi: 10.1038/srep38576 (2016).

**Publisher's note:** Springer Nature remains neutral with regard to jurisdictional claims in published maps and institutional affiliations.

## Supplementary Material

Supplementary Information
