# Supplementary material for: Designing a Broadband Pump for High-Quality Micro-Lasers via Modified Net Radiation Method
Source: Sci Rep. 2016 Dec 7;6:38576. doi: 10.1038/srep38576 (PMC5141496; doi:10.1038/srep38576)
Supplement: Supplementary Information [file srep38576-s1.pdf]

SUPPLEMENTARY: Designing a Broadband Pump for High-Quality Micro-Lasers via Modified Net Radiation Method

SERGEY NECHAYEV[1], PHILIP D. REUSSWIG[2], MARC A. BALDO[2], CARMEL ROTSCHILD[1*]

[1]Department of Mechanical Engineering and Russell Berrie Nanotechnology Institute, Technion-Israel Institute of Technology, Haifa 32000, Israel

[2]Department of Electrical Engineering and Computer Science, Massachusetts Institute of Technology, 77 Massachusetts Avenue, Cambridge, MA 02139, USA

**Corresponding authors: carmelr@technion.ac.il*

I-Net radiation method for solar light absorption

We utilize the net radiation method[1], which is a convenient tool for addressing incoherent light absorption in thin films, to calculate the solar light absorption in a stratified medium. The incoherent light approximation remains valid as long as the source coherence length is smaller than the optical path in the sensitizer layer, $l_c^{sun} \leq Re(n_s)\frac{t_s}{cos\,(\theta)}$, where l_c^{sun} is the solar light coherence length, t_s is the thickness of the sensitizer length, θ is the angle of propagation of the light in the sensitizer, and n_s is its refractive index. This condition holds true for layers that are thicker than 350 nm because $l_c^{sun}{\sim}0.6\mu m, Re(n_s) = 1.7$.

All of the calculations are presented for 2-layer structures for clarity, and they are easily extended to any number of layers by utilizing the more general equations that are presented in the manuscript. In the manuscript, the simulations are performed for 4-layer devices with non-absorptive cladding (as depicted in Fig. **1a** in the manuscript).

Consider the structure in Figure **S1** and the media enclosed between planes *i* and *i+1*. In the net radiation method, the optical field intensity is defined as the sum of the forward and backward propagating intensity waves. At the *i*th plane, the outgoing and incoming intensities are designated as $J_i^{\pm}(\omega,\theta)$ and $G_i^{\pm}(\omega,\theta)$, respectively, where ω,θ stand for the angular frequency and angle of incidence, respectively. Planar systems have axial symmetry, and therefore, θ measured in any layer defines the angle in all of the other layers, according to Snell's law. Our sign convention is that " $+$ " defines intensity components that are situated in the medium above the interface, and" $-$ " is for the medium below the interface. In addition, in each medium, the incoming and outgoing intensity components are connected by the equations $G_i^{+} = T_{i,i+1}J_{i+1}^{-}, G_{i+1}^{-} = T_{i,i+1}J_i^{+}$ via the transmittance $T_{i,i+1}$ of the layer between planes *i* and *i+1*.

First, we start with the case of solar light absorption in the sensitizer layer. In this case, the boundary conditions correspond to the solar flux at normal incidence from the positive direction of the z-axis. $G_0^{-} = I_{sun}(\omega,\theta), G_2^{+} = 0$, where $I_{sun}(\omega,\theta)$ is the solar flux per frequency. First, we write the equations that connect the inbound intensities with the outbound intensities through the specular reflectance coefficients.

$$J_0^{-} = R_sG_0^{-} + (1-R_s)G_0^{+} = R_sI_{sun}(\omega) + (1-R_s)G_0^{+}$$
$$J_0^{+} = (1-R_s)G_0^{-} + R_sG_0^{+} = (1-R_s)I_{sun}(\omega) + R_sG_0^{+}$$
$$J_1^{-} = R_{sg}G_1^{-} + \left(1-R_{sg}\right)G_1^{+}$$
$$J_1^{+} = (1-R_{sg})G_1^{-} + R_{sg}G_1^{+}$$
$$J_2^{-} = R_gG_2^{-} + \left(1-R_g\right)G_2^{+} = R_gG_2^{-}$$
$$J_2^{+} = (1-R_g)G_2^{-} + R_gG_2^{+} = (1-R_g)G_2^{-}$$

$R_s(\theta,\lambda), R_g(\theta,\lambda), R_{sg}(\theta,\lambda)$, are the polarization-dependent Fresnel's reflectance coefficients at the air-sensitizer, air-gain media and sensitizer-gain media interfaces, respectively and λ is a free space wavelength. Next, the relations between the inbound and outbound components through the transmittance are used to eliminate the outbound components:

$G_0^+ = T_s J_1^- \ ; \ G_1^- = T_s J_0^+$
$G_1^+ = T_g J_2^- = J_2^- \ ; \ G_2^- = T_g J_1^+ = J_1^+$

Here, the transmittance of the gain medium $T_g(\lambda, \theta_g) = 1$, i.e., we neglect the direct pump absorption in the gain media owing to its expected thickness (on the order of several microns) and low gain medium absorption coefficient $\alpha_g(\lambda)$ in the visible spectral range. The sensitizer absorption follows an exponential Beer-Lambert Law at normal incidence, and thus, sensitizer transmittance $T_s = e^{-\alpha_s(\lambda)t_s}$, where $\alpha_s(\lambda)$ is the absorption constant of the sensitizer. We rewrite the equations for the $J_i^\pm$ components:

$$J_0^- = R_s I_{sun}(\theta) + (1 - R_s)T_s J_1^-$$
$$J_0^+ = (1 - R_s) I_{sun}(\theta) + R_s T_s J_1^-$$
$$J_1^- = R_{sg} T_s J_0^+ + (1 - R_{sg}) J_2^-$$
$$J_1^+ = (1 - R_{sg}) T_s J_0^+ + R_{sg} J_2^-$$
$$J_2^- = R_g J_1^+$$
$$J_2^+ = (1 - R_g) J_1^+$$

Because we are interested in only 4 of the 6 components, we can rewrite them in a convenient linear equation form:

$$\boldsymbol{y} = \boldsymbol{A} \cdot \boldsymbol{x} + \boldsymbol{b}$$

$$\begin{pmatrix} J_0^+ \\ J_1^- \\ J_1^+ \\ J_2^- \end{pmatrix} = \begin{pmatrix} 0 & R_s T_s & 0 & 0 \\ R_{sg} T_s & 0 & 0 & (1 - R_{sg}) \\ (1 - R_{sg}) T_s & 0 & 0 & R_{sg} \\ 0 & 0 & R_g & 0 \end{pmatrix} \begin{pmatrix} J_0^+ \\ J_1^- \\ J_1^+ \\ J_2^- \end{pmatrix} + \begin{pmatrix} (1 - R_s) I_{sun}(\theta) \\ 0 \\ 0 \\ 0 \end{pmatrix}$$

The resulting equations are solved to obtain the absorbed photon flux in the sensitizer $Abs_s(\lambda, \theta, t_s, t_g)$ per angle of incidence per wavelength. Note that $\theta = 0$ for low solar concentrations, and the gain medium thickness t_g is irrelevant because $T_g(\lambda, \theta_g) = 1$ in the visible spectral range. The resulting absorbed solar photon flux in the sensitizer per wavelength when the sensitizer is pumped by non-concentrated solar illumination is:

$$Abs_s^{sol}(\lambda, t_s) = (1 - T_s)(J_0^+ + J_1^-) = I_{sun}(1 - R_s)(1 - T_s) F_{sun}$$

$$F_{sun} = \frac{1 + R_{sg} T_s - R_{sg} R_g + R_g T_s - 2 R_{sg} R_g^2 T_s}{1 - R_{sg} R_g - R_{sg} R_s R_g T^2{}_s - R_s R_g T^2{}_s + 2 R_{sg} R_s R_g T^2{}_s}$$

Because $R_{sg} \approx R_s \approx R_g \approx 0$ and the polarization is degenerate, we can approximate F_{sun} as

$$F_{sun} \approx 1 + (R_{sg} + R_g)T_s \approx 1$$

$$Abs_s^{sol}(\lambda, t_s) \approx I_{sun}(\lambda)(1 - R_s)(1 - T_s) \approx I_{sun}(\lambda)(1 - R_s)\left(1 - e^{-\alpha_s(\lambda)t_s}\right)$$

In other words, for the normal incidence, the non-reflected part of the solar radiation is absorbed according to the Beer-Lambert Law with the sensitizer absorption constant $\alpha_s(\lambda)$.

To obtain the total absorption in terms of the photon flux, $Abs_s^{sol}(\lambda, t_s)$ is integrated over the solar spectrum with respect to the wavelength.

$$Abs_s^{sol}(t_s) = \int Abs_s^{sol}(\lambda, t_s) d\lambda$$

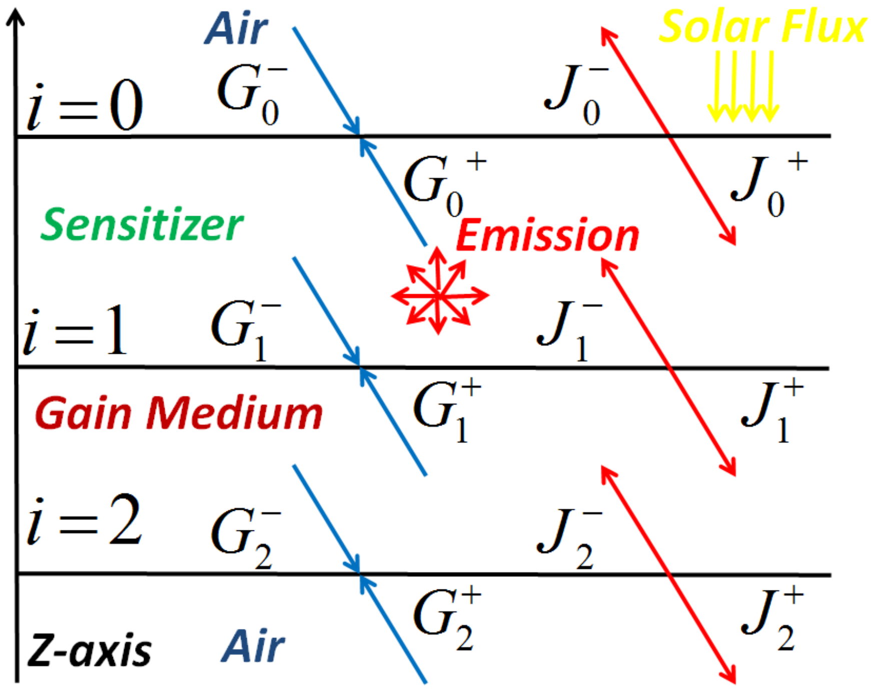

Figure S1 A schematic of the radiation net transfer at the interfaces between the different layers. The radiation at each interface on either side is modeled as the sum of the incoming and outgoing intensities impinging at a specific angle, polarization and frequency. The former are connected through Fresnel reflectance, transmittance and Snell's law. The boundary conditions are defined by the nature of the excitation. The $J_i^{\pm}, G_i^{\pm}$ values are obtained as a solution of the linear equation system from which the absorption in each layer can be deduced after integration over all of the angles and frequencies for the *s,p* polarizations separately.

II-Modified net radiation method for the absorption of the luminescence in the gain medium

After the excitation profile within the luminescent sensitizing layer was determined, the absorption of the luminescence in the gain media and self-absorption of the sensitizer can be defined. Assuming that there is no direct pumping of the gain media, no light is impinging on the structure from either side, i.e., $G_0^- = 0, G_2^+ = 0$; instead, the light is generated within the sensitizer layer. The sensitizer emission is then modeled as uniform non-polarized over angle of

incidence in the sensitizer θ_s in the range $0 \le \theta_s \le \frac{\pi}{2}$ with two normalization conditions (see the following chapters for a detailed explanation of θ_s). (**a**) The integration on the emission of the sensitizer, $I_{sens}(\omega, \theta_s)$, over the frequency, polarization and angles $0 \le \theta_s \le \frac{\pi}{2}$ in the sensitizer must correspond to the total absorbed solar photon flux multiplied by the sensitizer quantum efficiency η_s. (**b**) The line-shape of $I_{sens}(\omega, \theta_s)$ follows the sensitizer emission. The worst case approximation would be to assume that the luminescent light is uniformly emitted in the immediate vicinity of the top surface, which accounts for maximal self-absorption (the uniform emission is not a limitation of the model, and non-uniform emission can be easily accounted for by forcing the dependence of $I_{sens}(\omega, \theta_s)$ on θ_s as explained in the following section). In this case, the equations for G_0^+, J_0^+ are modified:

$G_0^+ = T_s J_1^- + luminescense = T_s J_1^- + \frac{1}{2} I_{sens}(\omega, \theta_s)$,

$J_0^+ = \left(1 - R_s^{s,p}\right) G_0^- + R_s^{s,p} G_0^+ + luminescense = \left(1 - R_s^{s,p}\right) G_0^- + R_s^{s,p} G_0^+ + \frac{1}{2} I_{sens}(\omega, \theta_s)$

where $R_s^{s,p}$ is the polarization-dependent reflectance coefficient at the air-sensitizer interface. The transmittance in each layer follows the Beer-Lambert Law with a propagation distance that depends on the angle of propagation in that medium: $T_s = \exp\left(-\alpha_s \frac{t_s}{cos\theta_s}\right), T_g = \exp\left(-\alpha_g \frac{t_g}{cos\theta_g}\right)$. Here θ_s, θ_g are the real-valued modeled angles of incidence in the sensitizer and gain media layers, respectively, relative to the optical axis.

The resulting equations are

$J_0^- = R_s G_0^- + (1 - R_s) G_0^+$

$J_0^+ = (1 - R_s) G_0^- + R_s G_0^+ + luminescence$

$J_1^- = R_{sg} G_1^- + \left(1 - R_{sg}\right) G_1^+$

$J_1^+ = (1 - R_{sg}) G_1^- + R_{sg} G_1^+$

$J_2^- = R_g G_2^- + \left(1 - R_g\right) G_2^+$

$J_2^+ = (1 - R_g) G_2^- + R_g G_2^+$

Substituting all of the above for G_0^+, J_0^+ and $G_0^- = 0, G_2^+ = 0$ results in

$$J_0^- = (1 - R_s)\left(T_s J_1^- + \frac{1}{2} I_{sens}\right)$$

$$J_0^+ = R_s\left(T_s J_1^- + \frac{1}{2} I_{sens}\right) + \frac{1}{2} I_{sens}$$

$$J_1^- = R_{sg} T_s J_0^+ + \left(1 - R_{sg}\right) J_2^- T_g$$

$$J_1^+ = (1 - R_{sg}) T_s J_0^+ + R_{sg} J_2^- T_g$$

$$J_2^- = R_g J_1^+ T_g$$

$$J_2^+ = (1 - R_g) J_1^+ T_g$$

Rewriting 4 of the above 6 equations in the matrix form $\boldsymbol{y} = \boldsymbol{A} \cdot \boldsymbol{x} + \boldsymbol{b}$, we obtain

$$\begin{pmatrix} J_0^+ \\ J_1^- \\ J_1^+ \\ J_2^- \end{pmatrix} = \begin{pmatrix} 0 & R_s T_s & 0 & 0 \\ R_{sg} T_s & 0 & 0 & (1-R_{sg})T_g \\ (1-R_{sg})T_s & 0 & 0 & R_{sg}T_g \\ 0 & 0 & R_g T_g & 0 \end{pmatrix} \begin{pmatrix} J_0^+ \\ J_1^- \\ J_1^+ \\ J_2^- \end{pmatrix} + \begin{pmatrix} \frac{I_{sens}}{2}(1+R_s) \\ 0 \\ 0 \\ 0 \end{pmatrix}$$

$$Abs_s^{1sun}(\lambda, \theta, t_s, t_g) - = (1 - T_s)(J_0^+ + J_1^-) = \frac{I_{sens}}{2}(1 + R_s)(1 - T_s)F_s(\theta)$$

$$Abs_g^{1sun}(\lambda, \theta, t_s, t_g) - = (1 - T_g)(J_1^+ + J_2^-) = \frac{I_{sens}}{2}(1 + R_s)(1 - T_g)F_g(\theta)$$

Here $Abs_s^{1sun}(\lambda, \theta, t_s, t_g)$ is the self-absorption of the sensitizer, i.e., absorption of the sensitizer emission in the sensitizer layer itself, when the sensitizer is subject to non-concentrated solar irradiation. $Abs_g^{1sun}(\lambda, \theta, t_s, t_g)$ is the absorption of the sensitizer emission in the gain medium, when the sensitizer is subject to non-concentrated solar irradiation.

$$F_s(\theta) = \frac{1 - R_{sg}R_g T^2{}_g + T_s(R_{sg} + R_g T^2{}_g - 2R_{sg}R_g T^2{}_g)}{1 + 2R_{sg}R_s R_g T^2{}_s T^2{}_g - R_{sg}R_s T^2{}_s - R_{sg}R_g T^2{}_g - R_s R_g T^2{}_s T^2{}_g}$$

$$F_g(\theta) = \frac{(1 - R_{sg})T_s(R_g T_g + 1)}{1 + 2R_{sg}R_s R_g T^2{}_s T^2{}_g - R_{sg}R_s T^2{}_s - R_{sg}R_g T^2{}_g - R_s R_g T^2{}_s T^2{}_g}$$

To gain intuition, we next examine the trapped light at angles above the critical angle: In this case $R_s = R_g = 1, R_{sg} \approx 0$ and:

$$F_s(\theta) \approx \frac{1 + T_s T_g^2}{1 - T_s^2 T_g^2} \qquad F_g(\theta) \approx \frac{T_s(T_g + 1)}{1 - T_s^2 T_g^2}$$

The absorption in each layer is then given by

$$Abs_s^{1sun} \approx I_{sens}(1 - T_s)\frac{1+T_s T_g^2}{1-T_s^2 T_g^2} \qquad Abs_g^{1sun} \approx I_{sens}(1 - T_g)\frac{T_s(T_g+1)}{1-T_s^2 T_g^2}$$

The pump efficiency, which is the ratio between the gain media absorption and self-absorption, can be defined as

$$Abs_g^{1sun} \Big/ Abs_s^{1sun} \approx \frac{(1 - T_g)}{(1 - T_s)} \frac{T_s(T_g + 1)}{1 + T_s T_g^2} \sim \frac{(1 - T_g)}{(1 - T_s)} \sim \frac{\alpha_g t_g}{\alpha_s t_s}$$

As expected, the sum of the absorbed photon flux equals the excitation flux:

$$Abs_g^{1sun}(\theta, \lambda) + Abs_s^{1sun}(\theta, \lambda) \approx I_{sens}(\theta, \lambda)$$

To obtain the total absorption in each layer, $Abs_x^{1sun}(\theta, \lambda)$ are integrated over the emission of the sensitizer, polarization and angle $0 \leq \theta_s \leq \frac{\pi}{2}$.

III-Modeling the emission of the sensitizer, including the directional emission and polarization

Because we discriminate between the forward and backward direction of emission, the full solid angle is 2π. Utilizing the Jacobian in spherical coordinates with full azimuthal symmetry, we can

write the normalization condition for the sensitizer

$$\frac{1}{2\pi}\sum_{s,p\ polarization}\int_{emission}\int_0^{\pi/2}\int_0^{2\pi} I_{sens}(\lambda,\theta_s) sin\theta_s\, d\varphi d\theta_s d\lambda$$

$$=\sum_{s,p\ polarization}\int_{emission}\int_0^{\pi/2} I_{sens}(\lambda,\theta_s)\, sin\theta_s\, d\theta_s d\lambda = \eta_s Abs_{sens}$$

Here φ is the azimuthal angle in the sensitizer and Abs_{sens} is the absorbed pump flux in the sensitizer. Therefore, the radiation emitted into the angle $d\theta_s$ for each polarization is $\frac{1}{2}I_{sens}(\lambda,\theta_s)sin\theta_s$, where $\frac{1}{2}$ stands for the uniform distribution of polarizations and $0 \le \theta_s \le \frac{\pi}{2}$. Choosing isotropic emission, we can write that $I_{sens}(\lambda,\theta_s) = \eta_s Abs_s^{1sun} f(\lambda)$, where $f(\lambda)$ is the normalized emission of the sensitizer, i.e., $\int f(\lambda)d\lambda = 1$. In the general case, as long as the normalization conditions hold for $I_{sens}(\lambda,\theta_s)$, we can choose it to describe the emission with various angles and spectral distributions.

For instance, in order to describe oriented dipoles emission, titled with angle θ_d relative the z-axis, one may assume $I_{sens}(\lambda,\theta_s) = I_0\, sin^2(\theta_s-\theta_d)f(\lambda)$, where I_0 is a constant defined by normalization condition:

$$\sum_{s,p\ polarization}\int_{emission}\int_0^{\pi/2} I_0\, sin^2(\theta_s-\theta_d)f(\lambda)\, sin\theta_s\, d\theta_s d\lambda = \eta_s Abs_{sens}$$

Integrating over θ_s,λ we obtain $\sum_{s,p\ polarization} I_0\left(\frac{1}{3}+\frac{1}{3}cos^3\theta_d - \frac{1}{3}sin2\theta_d\right) = \eta_s Abs_{sens}$ which is used to define I_0. This approach also allows to describe polarized emission with coupling between the spectral properties and the spatial distribution in which $I_{sens}(\lambda,\theta_s)$ is not separable function of λ,θ_s, as in the case of combination of different dyes with different orientations.

IV-Modeling the complex angle of incidence

In absorbing medium, the refractive index has a non-zero imaginary part. Therefore, the angle of incidence is complex and no longer represents the direction of propagation. However, most optical materials are weakly absorbing, i.e., $\left|{Im(n_s)}/{Re(n_s)}\right| = \left|{\kappa_s}/{Re(n_s)}\right| \ll 1$, where $\kappa_s = -Im(n_s) = \lambda\frac{\alpha_s}{4\pi}$ is the extinction coefficient of the sensitizer. In this case, the real part of the angle of incidence can be used as an approximation for the direction of the Poynting vector and, hence, the direction of the wave propagation. This condition holds true as long as $\lambda\alpha_s < 1$, or $\alpha_s < 10^6 m^{-1}$ at the sensitizer emission wavelength. We imply that the transverse component of the wave-vector is real[2-6], i.e., $Im(n_s k_0 sin\theta_s^c)$, where k_0 is the free space wavenumber and θ_s^c is the proper complex-valued angle of incidence in the sensitizer. This arrangement requires a correction for our choice of real θ_s by setting $sin\theta_s^c = sin\theta_s + i\gamma_s$, where $\gamma_s = \frac{\kappa_s}{Re(n_s)}sin\theta_s$ is the

imaginary part of $sin\theta_s^c$. By Snell's law, we find that $n_g sin\theta_g^c = n_s sin\theta_s^c$, where θ_g^c is the proper complex-valued angle of incidence in the gain medium and n_g is the complex refractive index of the gain medium, from which the real angle of incidence in the gain media is deduced: $sin\theta_g = Re\left(sin\theta_g^c\right) = Re\left(\frac{n_s}{n_g} sin\theta_s^c\right)$. In this case, we have $cos\theta_s = \sqrt{1 - sin^2\theta_s}$, $cos\theta_g = \sqrt{1 - sin^2\theta_g}$. Utilizing this approach, the single path absorption is calculated by the Beer-Lambert Law as $T_g(\lambda, \theta_g) = exp(-\alpha_g t_g / cos\theta_g) = exp\left(-\alpha_g t_g / \sqrt{1 - sin^2\theta_g}\right)$ and $T_s(\lambda, \theta_s) = exp(-\alpha_s t_s / cos\theta_s) = exp\left(-\alpha_s t_s / \sqrt{1 - sin^2\theta_s}\right)$.

V-Second-order absorption and re-emission effects

We treat the absorption and re-emission events in a similar way as for luminescent solar concentrators[7]. Noting that the rate of absorbed photons in each layer is proportional to the overall sensitizer emission $Abs_g^{1sun}, Abs_s^{1sun} \propto I_{sens}$, we can define the fraction of absorbed sensitizer emission in each layer by $Pabs_g = {Abs_g^{1sun}}/{I_{sens}}$, $Pabs_s = {Abs_s^{1sun}}/{I_{sens}}$. These values define the probability of the photon emitted by the sensitizer to be absorbed either in the sensitizer $Pabs_s$ or in the gain media $Pabs_g$. Each photon that is absorbed in the sensitizer is re-emitted with a probability of η_s and can then be absorbed in the gain media with a probability of $Pabs_g$. Therefore, the rate at which photons are absorbed in the gain media is a result of the sum of the infinite series of first (I), second (II) and higher order emission events, i.e., $Pabs_g^{tot} = Pabs_g + (Pabs_s\eta_s)^1 Pabs_g + (Pabs_s\eta_s)^2 Pabs_g + \cdots = Pabs_g \sum_{i=0}^{\infty} (Pabs_s\eta_s)^i = \frac{Pabs_g}{1 - Pabs_s\eta_s}$, where $Pabs_g^{tot}$ is the probability of the photon that is emitted by the sensitizer to be absorbed in the gain medium after all of the absorption and re-emission events. These secondary effects become significant when the self-absorption and quantum efficiency are both high.

VI – Guided modes loss

Additionally, in the thin planar waveguide, the lasing mode tail overlaps with the high absorption region of the sensitizer – Figure **S2a** presents the fundamental (blue line) and first mode (red line) intensity in a planar waveguide with a core thickness of $t_{core} = 2\ \mu m$, wavelength $\lambda = 1064\ nm$, indices $n_{core} = 1.82$ and $n_{clad} = 1.7$ of the core and cladding, respectively. Figure **S2b** shows how the mode intensity propagating in the cladding region decreases with the core thickness (blue dashed and red dashed lines for the fundamental and first mode, respectively, left logarithmic axis), and how it affects the resonator Q-factor if the losses in the cladding are orders of magnitude higher than in the core. The perturbation theory for a low-loss approximation[8]

gives a simple power average expression for a total attenuation coefficient $\alpha = \sum \alpha_i P_i / \sum P_i$. Here, P_i is the fraction of the total power in each region obtained from the modal solution, and α_i is the corresponding attenuation coefficient, from which a Q-factor is deduced as $Q = \tau_c \nu_L$, where ν_L is the lasing frequency and τ_c is the photon lifetime in the cavity. The coherence time τ_c[9-11] is given by $\tau_c^{-1} = \frac{c}{n}\left(\alpha - \frac{1}{l} ln\sqrt{R_{oc}}\right)$, with n being an effective refractive index of the guided mode and R_{oc} is the output coupling mirror's reflectivity. As an example, Figure **S2b** shows the resulting Q-factor for $\alpha_{core} = 0.3m^{-1}$, $\alpha_{clad} = 10^3 m^{-1}$ (blue solid and red solid line for the fundamental and first mode, respectively, right logarithmic axis). As observed, even if a small fraction of the power propagates in the sensitizer, the Q-factor is limited; therefore, it is constructive to induce a spatial separation between the sensitizer and the gain medium layers. In contrast to near-field sensitization, radiative energy transfer allows avoiding this negative effect by introducing lossless cladding with an intermediate refractive index between the sensitizer and gain medium (as shown in Figure **1a**), which effectively confines the lasing mode in the low loss region and increases the Q-factor to the value of the unperturbed cavity. For this case, the results obtained in Figure **2** in the manuscript are valid as all simulations assumed lossless cladding with intermediate refractive index $n_{clad} = 1.75$.

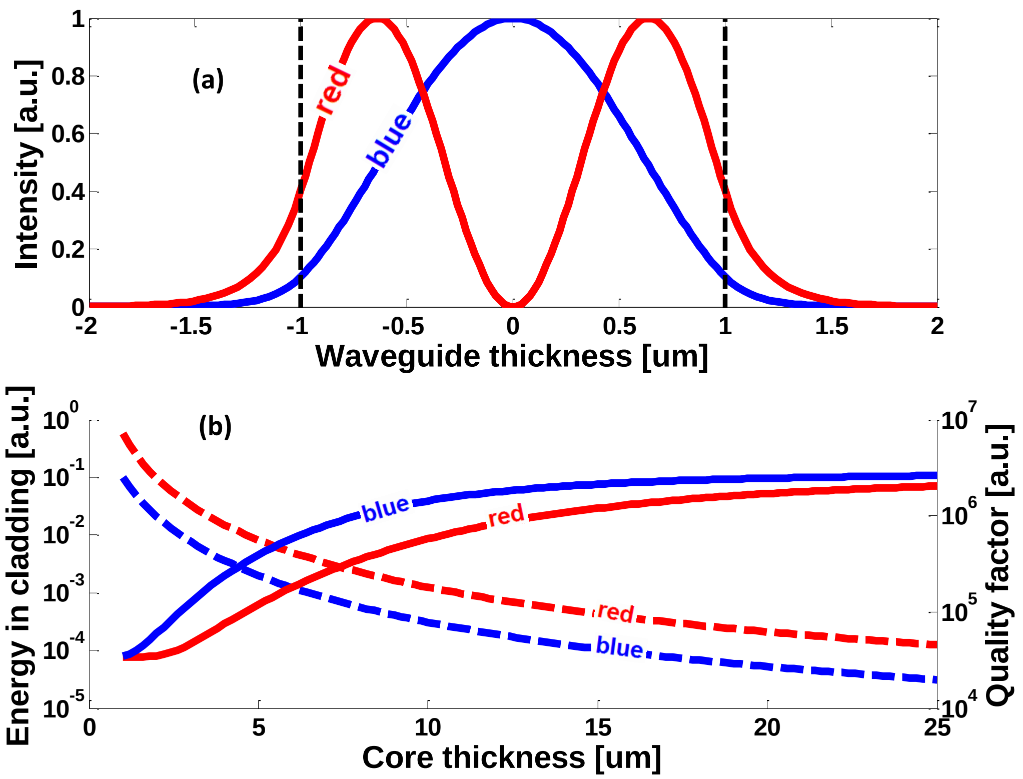

Figure S2 (**a**) Intensity profile of guided modes in a symmetric slab waveguide with indices $n_{core} = 1.82$, $n_{clad} = 1.7$ of the core and cladding, respectively, for the core thickness $t_{core} = 2\mu m$ (marked as vertical dashed black lines) and wavelength $\lambda_L = 1064\ nm$. The fundamental and first modes are shown in blue and red lines, respectively. (**b**) Fraction of the mode energy that propagates in the cladding as a function of the core thickness for the fundamental mode and first mode (blue and red dashed lines, respectively, left logarithmic axis), and the resulting attenuation of the Q-factor for the fundamental and first mode (blue and red solid lines, respectively, left logarithmic axis) for $\alpha_{core} = 0.3\ m^{-1}$, $\alpha_{clad} = 10^3\ m^{-1}$.

VII-Sensitizer Intensity Response

To verify that AlQ_3:DCJTB(2%):Pt(TPBP)(4%) makes it possible to construct a SPL we tested glass slides with deposited AlQ3:DCJTB(2%):Pt(TPBP)(4%) under various solar concentrations. Red line in **Figure S3** shows that the luminescent output of the sample starts to decrease already at concentrations above 1 sun. The values in blue line of **Figure S3** are normalized to the linear extrapolation of the response obtained under very low solar concentrations.

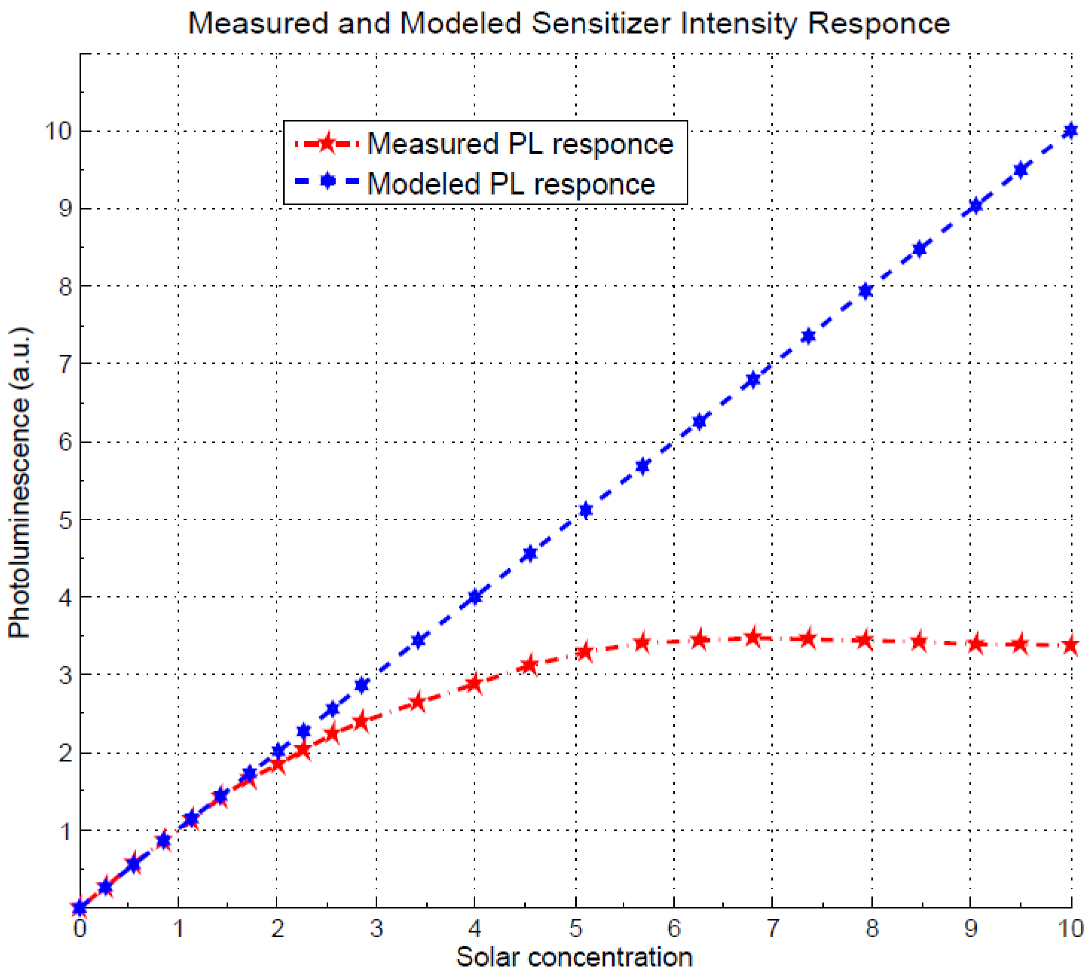

Figure S3 Linear extrapolation of the intensity response of AlQ_3:DCJTB(2%):Pt(TPBP)(4%) obtained at very low solar concentrations (blue) and actual behavior of this organic complex when illuminated with solar radiation at different concentrations.

References

1. Siegel, R. Net radiation method for enclosure systems involving partially transparent walls. (1973).
2. Orfanidis, S. J. Electromagnetic Waves and Antennas. (§7.9). Available at: http://www.ece.rutgers.edu/~orfanidi/ewa/.
3. Parmigiani, F. Some aspects of the reflection and refraction of an electromagnetic wave at an absorbing surface. Am. J. Phys. **51,** 245–247 (1983).
4. Roo, R. D. & Tai, C.-T. Plane wave reflection and refraction involving a finitely conducting medium. IEEE Antennas Propag. Mag. **45,** 54–61 (2003).
5. Chang, P. C. Y., Walker, J. G. & Hopcraft, K. I. Ray tracing in absorbing media. J. Quant. Spectrosc. Radiat. Transf. **96,** 327–341 (2005).
6. Dupertuis, M. A., Acklin, B. & Proctor, M. Generalization of complex Snell–Descartes and Fresnel laws. J. Opt. Soc. Am. A **11,** 1159 (1994).
7. Batchelder, J. S., Zewai, A. H. & Cole, T. Luminescent solar concentrators. 1: Theory of operation and techniques for performance evaluation. Appl. Opt. **18,** 3090–3110 (1979).
8. Adams, M. J. Loss calculations in weakly-guiding optical dielectric waveguides. Opt. Commun. **23,** 105–108 (1977).
9. Grivas, C. Optically pumped planar waveguide lasers, Part I: Fundamentals and fabrication techniques. Prog. Quantum Electron. **35,** 159–239 (2011).
10. Grivas, C. Optically pumped planar waveguide lasers: Part II: Gain media, laser systems, and applications. Prog. Quantum Electron. **45–46,** 3–160 (2016).
11. Yariv, A. Photonics : optical electronics in modern communications /. (Oxford University Press, 2007).
